# Scale Matters: Temporality in the Perception of Affordances

**DOI:** 10.3389/fpsyg.2020.01188

**Published:** 2020-06-16

**Authors:** Melina Gastelum

**Affiliations:** Faculty of Philosophy and Literature (FFYL), National Autonomous University of Mexico (UNAM), Mexico City, Mexico

**Keywords:** temporality, affordances, ecological psychology, enactivism, temporal scales, synergies

## Abstract

In this paper I seek to unify enactive and ecological approaches to cognitive science by emphasizing the fact that both approaches view cognitive processes as being inherently temporally extended. My hypothesis is that characterizing the temporal scales in which perception of affordances occur, they can serve different purposes of explanation within the theories. Specifically, the paper brings together, on the one hand, Chemero’s (2009) dynamicist understanding of affordances, which he called affordances 2.0, with, on the other hand, a distinction originally made by Varela (1999), and later taken up by Shaun Gallagher (2011, 2017b), between three different timescales for understanding cognition: the elementary, the integrative, and the narrative. Varela’s three-fold distinction was originally intended as a way of identifying phenomenological events as being causally coupled to specific cellular events happening within the nervous system. The central claim of the present paper is that affordances, likewise, should be understood in terms of these three different timescales. I show that these temporal scales can be a useful toolkit for explaining the perception and learning of affordances and at the same time unifying enactivism and ecological psychology claiming that affordances serve a different explanatory role depending on which time scale you consider them at. If you are interested in explaining the embodied assemblies that form the always changing sensorimotor contingencies, then you see the elementary scale. If you’re interested in explaining perception at the integrative scale, then affordances are solicitations that get actualized and bear an umwelt at that same scale. The perception of affordances as such is constituted by the integration of these first two scales, and the experience of it can be characterized by the husserlian structure of experience with its intrinsic temporality. Finally, if you are interested in explaining change in the animal-environment system over developmental time, that is, learning, then affordances are roughly what Chemero proposed and they operate at the narrative scale. But it is important to say that the three scales are always intertwined because learning and perception are ongoing processes that in many senses are impossible to separate. Finally, I discuss the importance of scales from the macro to micro levels for understanding behavior through affordances, considering them as synergies, where abilities and aspects of the environment are understood as constraints on the potential trajectories of such systems.

## Introduction

Embodied and situated approaches to cognition have gained terrain within cognitive science in the last decades. They have put forward questions that were traditionally neglected, such as the role of the body in cognition, and have opened a field of research alternative to representational approaches to the mind that is philosophically and operationally solid in its own right. Enactivism and ecological psychology have been without any doubt the most prominent and crucial approaches to this development. However, there has been a historical lack of compatibility between them. Although they share essential tenets about cognition and mind, their respective early formulations were explicitly critical to one another which has led them to emphasize different aspects of the mind in their specific terms: while enactivism focuses on the cognitive agent and its subjectivity, ecological psychology focuses on the environment and the relation that animals establish with it (Baggs and Chemero, 2018). In the last decade, it has been recognized that these approaches are not so distant after all, and even more, that bringing them together could be greatly fruitful for a solid radically embodied approach to cognition. In this paper I join this claim by analyzing the temporally extended character of the perception of affordances for both the approaches.

There are at least three points of agreement between enactivism and ecological psychology. First, they share a conception of perception that makes it continuous with action: for enactivists, perception is understood “as something we do” (Nöe, 2004; Heras-Escribano, 2019) in order to preserve our form of life (Di Paolo et al., 2017), while for ecological psychology, perception occurs in terms of invitations to act and it is “an achievement of the individual, not an appearance in the theater of his consciousness” (Gibson, 1979, p. 228).

Second, for both approaches the environment is constitutive of cognition: for enactivists, the environment is a constitutive element of adaptive interactions which are essential to sense-making (Di Paolo, 2005) and to maintain a central tension that gives autonomy to a living system. This autonomy emerges from a “primordial tension,” which refers to those dynamics by which the identity of an organism requires to persist while opening itself to the environment and making sense of it at the same time. Meanwhile, ecological psychology takes the organism-environment system as the unit of analysis of cognition as a whole, in other words, ecological psychology explains the way in which agents perceive information about affordances and relates to them. For both approaches, cognition doesn’t happen inside the head but in the interaction of an organism with its environment.

Third, both the enactive and the ecological psychology definitions of environment coincide in that the surroundings are primarily meaningful for the agent in an embodied, non-semantic, non-representational sense. The idea of sense-making in enactivism shows that the world is meaningful and constitutive inasmuch as it allows the organism to perform a certain action that is relevant for its autonomy, which is close to the ecological idea of affordance, that is, things are perceived meaningfully as they call for action (Costall, 1995, p. 470; Heras-Escribano, 2019, p. 23) and therefore the unit of analysis is the animal-environmental system as a coupled unity. In short, they are both embodied and situated approaches to cognition committed both with non-representationalism, with the continuity of perception and action and the end of the dualism between internal and external or agent and environment.

It might seem that not all conceptions of affordance proposed within ecological psychology are conceptually compatible with the cognitive agent proposed by enactive theory. For example, conceiving affordances as dispositional properties might seem like reducing cognitive agents’ engagement with the world to an almost mechanical response which directly contradicts the enactive conception of sense-making. I believe, however, in line with Baggs and Chemero (2018), that this conception of affordances as dispositions describes one of the various possible levels of analysis of the agent-environment engagement, namely, the total set of skills shared by agents with a common biological organization, a common niche and a common pattern of behavior, while the enactive approach describes a different level, namely, the individual level. In general, while the enactive account of agent-environment interaction in terms of sensorimotor schemes describes the configuration of the point of view of the cognitive agent, affordance theory focuses on the relational and dynamical properties that arise precisely in this sensorimotor interaction from the perspective of a population that shares a form of life, but I will explain this in the next section.

In this paper, the hypothesis is that by saying that environment is constitutive of cognition and adding that by understanding how the scales of temporality of the processes by which the engagement with it occurs (i.e., the perception of affordances) we can step forward in bringing together both approaches. For this, I claim that the conception of affordances 2.0 (Chemero, 2009) has to be taken seriously and further explained as a bridge between them. This involves taking the temporal dimension of abilities in affordances seriously, particularly in terms of interaction across multiple temporal scales. For this, I think that perception of affordances should be characterized in terms of dynamical agent-environment systems, with abilities and aspects of the environment (affordances 2.0) understood as constraints on the potential trajectories of such systems.

For this, in the first part of the paper I explain why temporal dynamics of affordances and affordance perception is incredibly important and how is it that Chemero (2009) proposed them. I also explain the division between physical world, habitat, and umwelt developed by Baggs and Chemero (2018) to understand the difference between objective and subjective affordances and the interplay between them, when they are potential and get actualized. In the second section I expand on the notion of intrinsic temporality of the perception of affordances, borrowing the husserlian structure of time consciousness and argue that it can be used to explain action/perception and experience of affordances which is a fundamental part of the temporal dynamics in them. In the third section, I introduce Varela’s (1999) three-fold distinction for neurodynamics and explain why, in my hypothesis, I think they can serve for characterizing the dynamisms of the perception and learning of affordances. I claim that differences in temporal scales is significant because they are constitutive of sensorimotor schemes dynamics, and sensorimotor schemes dynamics are constitutive of affordances, hence affordances. Henceforward, affordances are constituted by the abilities and the environment as relations that occur with an intrinsic temporality that is related to different temporal scales interconnected between them. These three scales are constitutive of the perception and learning of affordances whenever they are actualized and therefore whenever they bear an experience for the agent, so it might make sense to say that the entire organism-environment system retains changes in its structure as a result of the organism’s learning of sensorimotor contingencies. Finally, I discuss the importance on scales from the macro to micro levels of understanding behavior through affordances, considering them as synergies, where abilities and aspects of the environment are understood as constraints on the potential trajectories of such systems.

## Affordances 2.0, Habitats and Umwelt

### Affordances 2.0

In the ecological psychology tradition, there are at least three important assumptions at its basis: that perception is direct, that perception is for action, and that perception is of affordances (Chemero, 2009, p.98). As mentioned in the introduction, not all conceptions of affordances are compatible with the idea of bringing together ecological psychology and enactivism. Briefly put, there has been much discussion about how to characterize affordances. Some of Gibson’s followers have stated that affordances are dispositions in the environment (Turvey et al., 1981; Turvey, 1992) that get actualized through an effectivity from the individual that perceives them. I believe, however, that overcoming differences between the ecological and enactive theories has to do with the conceptualization of affordances you make and is essential for constructing a robust post-cognitivist framework in cognitive science, which is part of the aim of this paper. I believe Chemero’s conception of affordances 2.0 is an excellent departing point for that. As he claimed:

Combining Affordances 2.0 with enactivist studies of the organism makes radical embodied cognitive science a fully dynamical science of the entire brain–body–environment system: non-representational neurodynamic studies of the nervous system and sensorimotor abilities. (Chemero, 2009, p. 152)

And this is so because as Chemero has rightly noted, ecological psychologists usually “define affordances statically” (2009, p. 150). Consequently, as an attempt to dynamize the concept of affordances, he introduces the concept of affordances 2.0:

Affordances are relations between abilities to perceive and act and features of the environment (2009, p. 150). Affordances and abilities are not just defined in terms of one another as in the dispositional and relational views (…), but causally interact in real time and are causally dependent on one another. […] This reconceptualization of affordances is explicitly formulated to make the natural, but largely unmade, connections between ecological psychology and the enactivist movement in cognitive sciences. (Chemero, 2009, pp. 151–152)

The key point is to conceptualize affordances as interacting in real time, as dynamically coupling with the rest of the environment and among them. Silberstein and Chemero (2011, p. 7) expand on this saying that the animal’s endogenous system has endogenous dynamics that generates and constitute the sensorimotor abilities and the whole nervous system. The sensorimotor abilities are coupled with a specific niche which in turn modulates the dynamics of the nervous system. So, “affordances and abilities are not just defined in terms of one another, but causally interact in real time and are causally dependent on one another in a non-linear fashion” (Silberstein and Chemero, 2011, p. 8).

Affordances 2.0 are compatible with the enactive idea that agents actively make sense of their environment in terms of how it affects them, but how does this happen and in which temporal scales? I will discuss this in the next section.

### Environment, Habitat, and Umwelt

Another important conception that I will use in this paper is the difference between physical world, habitat, and umwelt that Baggs and Chemero (2018) recently developed. They used this distinction to clarify disagreements between ecological psychology and enactivists, as well as to clarify certain tensions within the former in relation to the conceptualization of affordances.

Gibson (1979) made a crucial distinction between the physical world and the environment of animals.

The physical world exists at all spatial and temporal scales, from nanoseconds and nanometers to millennia and galaxies. The animal’s environment is limited to the behavioral, middle scale. For humans, the spatial scale of the environment is from millimeters to kilometers and the temporal scales can be from hundreds of milliseconds to years. (Gibson, 1979, p. 12)

Gibson also pointed that the environment of animals is perceived in terms of ecological events, and not in terms of time; time is actually an abstraction. Ecological events have been characterized as “changes in the layout of affordances of the animal-environment system” (Chemero, 2000, p. 39). Notably, “the physical world is inherently meaningless, but the environment is not: it contains affordances” (Baggs and Chemero, 2018, p. 4).

The relevance of scales starts here: the ecological scale matters as I want to highlight in the title of the paper. This scale is crucial when explaining what cognition is because within it there’s clarity about the environment being a subset of the whole physical world, and therefore, the ecological scale sets up in space and time the *behavioral scale* for animals, where and when affordances occur. Because the environment contains affordances, there is no need for the animal to construct meaningful experiences by building or manipulating representations in some phenomenal realm, or even in the brain. “In fact, meaningful experience doesn’t happen inside the animal but as perceiving occurs: an animal using information to learn about the affordances of the environment is having meaningful experience” (Baggs and Chemero, 2018, p. 5).

Furthermore, Baggs and Chemero argue that it is necessary to subdivide what Gibson referred to as the environment. “We need to make a finer distinction between, (1) the environment as a set of resources for a typical, or ideal, member of a species, which we call the habitat, and (2) the environment as the meaningful, lived surroundings of a given individual, which we call the umwelt” (Baggs and Chemero, 2018, p. 6).

The environment in which perception/action is situated, that is, what appears meaningful to the agent and toward which she orients her actions, is called her umwelt. This concept was originally introduced by Jakob von Uexküll, and it captures the *phenomenal world* an organism inhabits and in which everything it will ever experience occurs. An umwelt arises from the coupling of one single organism, with its needs and physiological possibilities, to its physical environment through a functional cycle of perception and action (Uexküll Von, 1929, 1957). In the terminology of Baggs and Chemero, this would amount to a finer distinction, this would be the coupling with a particular habitat, not a physical world, because according to Gibson this later one is meaningless (it is the scale of the physical, not the behavioral). This means that the umwelt is not shared by a whole species nor does it consist in the physical world to which everything belongs; instead it belongs to each individual organism (although different umwelts can actually be quite similar for different individuals of the same species). Thus, in von Uexküll words, biologists consider “as many worlds as there are subjects” (1929, p. 70). An Umwelt is not exclusively constituted by the biological needs and possibilities of an individual: what appears meaningful to a human agent is largely constituted by her repertoire of acquired sensorimotor abilities. An aspect of the environment appears meaningful to an agent when it becomes part of what makes her action possible, that is, when it allows her carrying out networks of sensorimotor abilities; in other words, when it becomes a relation. This means that when sensorimotor abilities are learnt and carried out, new affordances (potential at one point in time, from the habitat) are incorporated in an individual’s umwelt. An individual’s umwelt is thus shaped by the action possibilities proper to the specific biological organism she is but also by her personal history of interactions with her habitat.

This distinction between physical world, habitat, and umwelt allows for distinguishing the individual’s set of affordances given its biological set up, its development, and her history (the umwelt) from the general (objective) set of affordances that are possible for a group of individuals or species (the habitat), the ones that could be seen as dispositions or potential affordances 2.0 (relational). In other words, there are affordances that exist objectively for an individual as a member of a species or of a group of individuals and some affordances that are perceived as soliciting for a given individual given its histories and interactions within that particular niche, and that become actualized and thus, form part of their ever-changing umwelt.

So, here we have two levels of description, one for particular agents and one for a group of individuals. Importantly, the conception of affordances is also dependent on these levels of description. Chemero argues that affordances 2.0 are relational and that they can give an account of learning and bodily capacities into the perceptual processing, which in turn can explain why some affordances are salient for a particular individual and not for others. On the other hand, the affordances as dispositions (Turvey, 1992) allows affordances to play a role in evolution but is not clear about how some affordances are actualized or are salient to a particular individual in a species. This is a tension also noticed by Reed:

An affordance is only a relation when an animal perceives or uses it, because then the animal comes into relationship with the relevant feature of its environment. Affordances in the animal’s niche are not relations; they are instead resources—in this case, resources for obtaining value from the environment. (Reed, 1996, p. 26)

So, the tension is solved with the distinction that Baggs and Chemero explained between habitat, where affordances are objective dispositions for the species that could or not invite behavior for particular individuals, and the relational affordances that entail an umwelt, where affordances depend on the particular history of development (ontogeny) and thus, are subjective and salient for a particular individual when we perceive and act upon them. Finally, it is important to notice that an individual’s umwelt can actually go beyond some particular habitat due to significant individual variations; here we can think in some perceptual variations, like autism, or even a more sociocultural habitat, like that of a particular culture or form of life.

### Enactivism in the Habitat and Umwelt

As we just saw in the last section, Gibson understood that the physical world as a whole is meaningless for the behavior of animals. When one considers it from the point of view of an idealized member of the species, “parts of it partly constitute a habitat of that species, and when one considers it from the point of view of an individual, parts of it partly constitute an umwelt” (Baggs and Chemero, 2018, p. 12).

The way to put this idea for phenomenologists and sensorimotor enactivists is by saying that the umwelt is given in experience, while ecological psychologists say it is perceived directly. This will be crucial for this paper, because this means that “given in experience” and “perceived directly” implies the same: that the access to the umwelt is not mediated by representations, but it is enacted, acted upon in the ongoing couplings with the environment. This connotes that is “brought forth” in the historic relation between the agent and the habitat. The umwelt then is not stable either, it changes by the history of structural coupling the individual goes through in her development. For enactivism, the world (meaning, intentionality) is not pre-given or predefined, but is structured by cognition and action, and shows up as an affordance space (Brincker, 2014; Gallagher and Lindgren, 2015) or a “landscape of affordances” (Rietveld and Kiverstein, 2014).

For enactivists, the role of environment is constitutive of cognition because of the way agency is shaped in the relationships with it. The role of the interaction of the organism-environment is explained through sensorimotor contingencies. Sensorimotor contingencies allow us to explain cognition in the way Varela et al. (1991, p. 173) put it: “perception consists in perceptually guided action.” The organism couples with the environment through a feedback relation between perception/action and sensations. The correspondence of action and incoming sensation establishes regularities, which are the contingencies, and when they get mastered by the agent and integrated with other contingencies, they become sensorimotor schemes and with time habits that allow to anticipate upcoming events and guide behavior. O’Regan and Noë (2001, p. 82) put it like this: “agency in a cognitive sense is understood as the skillful purposive behavior of the whole organism, and this skillfulness is based on the mastery of these sensorimotor regularities.”

Another notion in sensorimotor enactivism that enlightens the conception of umwelt is the idea of sense making. This concept explains the meaningful or valuable environmental aspects for the organism (Di Paolo, 2005). Sense making occurs as every agent establishes a particular history of interactions (sensorimotor contingencies) through repertoires of sensorimotor schemes and regulations (adaptivity) (Heras-Escribano, 2019, p. 9) that shape constantly her individual perspective in a particular habitat. This is also why we can say that the umwelt is never complete and it’s a continuous activity of the organism. This is the reason why enactivists also analyze affordances as objects of perception (Nöe, 2004; Di Paolo et al., 2017), because they share the idea of meaning as a product of the way in which the environment is related to the organisms’ capacities in an embodied and situated way. Baggs and Chemero (2018) explained that the umwelt “requires work,” which means, like in the enactivist approach, that the internal organization of the animal (the sensorimotor schemes that fill in the theory of sensorimotor contingencies) can explain how the animal is continuously making sense and adapting to its surroundings, in the process of shaping her continuously changing umwelt, and also, constituting her agency.

To conclude this section, we can resume that the definitions of environment for enactive and ecological approaches share that it is meaningful in an embodied, non-semantic, non-representational sense. For the enactivists, sense making permits explaining how the agents live in a meaningful world inasmuch as it allows the organism to perform certain actions, which also is in the core of ecological psychology with the idea of affordance, also called “the meaning of things for action” (Costall, 1995, p. 470).

## Husserl and the Structure of Temporality

As mentioned before, Chemero proposed affordances 2.0 to introduce dynamism into its conception. In this section I will develop an account of this dynamism, borrowing some conceptions from Husserl and importing them into perception of affordances.

The question is: How can we characterize the temporally extended character of the perception of affordances? We will adopt Husserl’s analysis of the intrinsic temporal structure of experience that can be applied not just to experience/consciousness but also to embodied action. Berthoz (2000), for example, suggested that the Husserlian analysis of the retentional-protentional structure of experience is a model that also works for the processes involved in motor control (Gallagher, 2011; Gallagher et al., 2017). This structure gives an intrinsic temporality to all motor actions. This is to say that nothing is an affordance if there is a knife-edge present, because at a single present moment, nothing would be afforded, because a temporal window for its realization is needed. If there wasn’t an intrinsic temporality in the actualization of affordances, if there weren’t anticipations in a set of possibilities in the world, we would never be able to perceive affordances.

Intrinsic temporality is something we can find in all the dynamics of bodily movement and action and manifests itself at both the subpersonal and the personal levels of analysis, which we will explain further on.

This intrinsic temporality is not objective time that can be measured by a clock, although action certainly does take place in time, and it may be important in various contexts that its duration can be measured. Phenomenologists distinguish objective time from lived time (e.g., Merleau-Ponty, 1962; Husserl, 1991). The latter is time as we experience it passing, sometimes seeming to pass slowly and sometimes rapidly. Intrinsic temporality includes more than lived or phenomenological time; it includes a temporal structuring that shapes action and experience. (Gallagher et al., 2017, p. 84)

With regard to action and motor control, this intrinsic temporality is expressed in Henry Head’s definition of the body schema. “According to Head, the body schema dynamically organizes sensory-motor feedback such that the final sensation of position is ‘charged with a relation to something that has happened before’ (Head, 1920, p. 606). Merleau-Ponty borrowing Head’s metaphor of a taximeter suggests that movement is organized according to the “time of the body, taximeter time of the corporeal schema” (Merleau-Ponty, 1968, p. 173). Body schematic processes incorporate past moments into the present” (Gallagher et al., 2017, p. 86):

At each successive instant of a movement, the preceding instant is not lost sight of. It is, as it were, folded into the present. Movement draws together, on the basis of one’s present position, the succession of previous positions, which envelop each other. (Merleau-Ponty, 1962, p. 140)

Reaching for an object, for example, involves feed-forward components that allow last-minute adjustments if the object is moved, and the grasp of my reaching hand tacitly anticipates the shape of the object to be grasped. “This is not blind automaticity since the grasp is shaped according to the specific intentional action involved (see MacKay, 1966; Wolpert et al., 1995; Jeannerod, 2001)”. Berthoz (2000, p. 25) suggests that anticipation is “an essential characteristic” of motor functioning. Similar anticipations characterize the sensory aspects of perception (see Wilson and Knoblich, 2005 for review) (Gallagher et al., 2017). Since these prospective processes are present generally, even in infants, the “conclusion that [anticipatory processes] are immanent in virtually everything we think or do seems inescapable” (Haith, 1993, p. 237).

**FIGURE 1:**
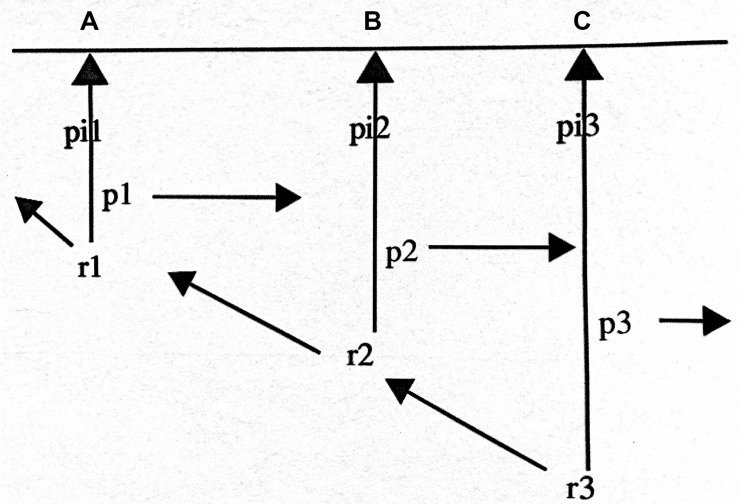
Husserl’s model of time consciousness, from Gallagher (1998, 2011) and Gallagher et al. (2017, p. 85).

Husserl’s model, called the *präsenzzeit*^1^, is represented in Figure 1. He applied this model to the conscious perception of a melody, but we will claim it can be applied to perception in general. It has three structural aspects: The horizontal line ABC represents a temporal object such as a melody of several notes. The vertical lines represent abstract momentary phases of an enduring act of consciousness.

Figure 1, from Gallagher (1998, 2011), and Gallagher et al. (2017, p. 85).

Each phase is structured by three functions:

• Primal impression (pi), allowing for the consciousness of an object (a musical note, for example) that is simultaneous with the current phase of consciousness;
• Retention (r), which retains previous phases of consciousness and their intentional content;
• Protention (p), which anticipates experience that is just about to happen.

It is important to say that one could think that Husserl saw consciousness as an internal metaphysical entity, but I think this is a misreading. Let’s consider this: “Consciousness is only as long as it is open to the world; therefore, there is no interiority or exteriority. There is only one intentional fabric that is indissolubly, that of consciousness and the world” (Bech, 2001, p. 54). In his phenomenological tradition, this amounts to saying that consciousness is the experience, the “pure” experience that happens in the perception of the world.

Husserl (1989) argued that to do phenomenology properly, that is, to attend to the experiences themselves leaving all preconceptions aside, one has to bracket questions about the world beyond the experiences, but he is talking about a pre-given world as an objective (scientific) phenomena. This experience (the umwelt), of course, cannot be bracketed; what is bracketed are the judgments and reflections about the world. So, we are talking about the same experience, the umwelt, that is going to be temporally structured by this *präsenzzeit*. In other words, for Husserl there can only be experience (in the sense just explained) because there is an intrinsic temporal structure that supports it, the *präsenzzeit*.

Although the specific experiential contents of this structure from moment to moment progressively change, at any given moment this three-fold retention-primal impression-protention (RIP) structure is present as a unified whole. In the current phase, simultaneous with C (in Figure 1), there is a retentioning (r3) of the previous phase of experience, and this just-past phase includes its own retentioning of the prior phase. This means that there is a retentional continuum—r3(r2[r1]), and so forth—stretching back over recent prior experience, on the order of seconds. There is also a double intentionality to this retentional aspect. Retention retains the prior phases of consciousness/experience (what Husserl called longitudinal intentionality), but since those phases include primal impressions of the then current notes (B and A, respectively) retention also retains the prior notes of the melody (what Husserl called transverse intentionality), in the sequential order in which we heard them, which generally reflects the order in which they were sounded. Imagine if that were not the case. If there were no retention of previous notes, then we would not hear a melody. Indeed, if our experience were of only one moment at a time without experiential connection to previous moments, it would be impossible to make sense of the world. (Gallagher et al., 2017, pp. 85–86)

Protention, in turn, provides consciousness/experience with an intentional sense that something more will happen. This protentional aspect allows for the experience of surprise. In listening to a familiar melody, there is some sense of what is to come, a primal expectation of the notes to follow. The content of protention is never completely determinate since the future itself is indeterminate. Indeed, in some cases the content of protention may approach the most general sense of “something (without specification) has to happen next” (Gallagher et al., 2017, pp. 85–86).

Summarizing, Husserl’s claim is that dynamism and flow have their origin in the retentional and protentional structure of temporally extended experience/consciousness and it is the relations between retention, primal impression, and protention which constitute the temporality of the flow of experience (Kiverstein and Arstila, 2013, p. 455).

In terms of affordances, to perceive affordances is to experience affordances, there is not a division in perceiving/experiencing, all perception implies an experience that is in itself dynamical and will include different temporal scales in its performance, as we will see in the next section. Putting it bluntly, perception/experiences of affordances also have a temporal flow that originates in the RIP structure.

## Temporal Scales in the Perception and Experience of Affordances

Bringing together the last two sections, one can say that experientially, the umwelt is given by affordances that “stand out” among the rest of the affordances in a habitat for a particular agent. These attractive or relevant affordances are described as soliciting, or inviting, behavior (Withagen et al., 2012; Rietveld and Kiverstein, 2014). And when this behavior happens, affordances occur in motion and entail an experience with an intrinsic temporality that has a RIP structure.

The soliciting character of these relevant affordances is the experiential equivalent of a bodily “action readiness” on the part of the skilled individual, which entails orientation toward and preparation for possibilities for future action (Rietveld et al., 2018).

Gibson noticed it:

A sequence of external stimuli or, at the very least, the rhythms of the observer’s body, provide a flow of change, and it is this we perceive rather than a flow of time as such. (Gibson, 1975, p. 299)

By considering the perceiver acting and moving in an environment, its own motions “span” the entire event. This enables the perceiver to apprehend, as Gibson called it, a sequential structure. “Sequences of events can be apprehended in their entirety as an agent is resonating to the entire motion. Nonetheless, this appropriate responsiveness to the motions of the world, and the apprehension of sequences (especially on large scales of coordination), needs to be learned” (Van Dijk and Withagen, 2016, p. 10). The implication is that affordances are not properties or universals, they are unique for every animal because they emerge within the relationship between the environment and the perceiver, which means that different layouts afford different behaviors for different animals. Also, that affordances are related to movement and action of each agent in her own cadences. This is relevant because it means that Gibson’s theory is related to ecological perception on the basis of agency and the actions performed by an agent or animal (Menatti et al., 2016, p. 11).

Action readiness is a form of anticipation: I am ready to do something ahead of me (and I am always aware of what I’ve done to be in the situation I am in the moment). This of course requires previous experiences. Once an ability is acquired after a history of training, practice and experience in an environment (Ingold, 2011), the relationship between body and environment is modified. There is a familiarity in the world for the individual, somethings that she is attuned to, and that propitiates a particular umwelt for her when certain affordances solicit action. At that moment the level of skill rises to the point where the individual is able to perceive and respond immediately to affordances in this new domain (Rietveld, 2012), so that the intrinsic temporality itself changes with respect to its own as skillful practices develop and sensorimotor schemes responses become faster and faster (we will come back to this in the last section). Furthermore, action and perception of agents with a past full of previous interactions always causes that the environment is never encountered ahistorically. “All acting and perceiving is done in a flow of activity that is continuous for living beings. For us human beings the fields of action, the engagements in which we find ourselves, have both personal and cultural histories. Our subjectivity is dependent on our intersubjectivity. Social activity mediates individual psychology but does so in a manner that is fundamental, not additional […] the environment in which we human beings live and act is cultural to its core” (McGann, 2014, p. 9).

Adding to the anticipatory temporal window, there’s a concept called intentional arc (Merleau-Ponty, 1962) which adds to the readiness of coping skills. The concept of intentional arc situates skills in recursive loops of action, built up over time. Drawing on Merleau-Ponty, Dreyfus defines the intentional arc as “the way our successful coping continually enriches the way things in the world show up” (Dreyfus, 2014, p. 107). In other words, the intentional arc is a “feedback loop in which our actions and projections are drawn out of us by the meaningful features of the world and, in turn, alter the way the world shows up as soliciting us.” In short, the intentional arc shows a relationship between meaningful appearances in the environment and their performance as achieved through practice and repetition. Here is where we are borrowing a concept from Husserl to suggest that perception of affordances have a “retentional” character, that is, they do not merely guide future activity but are also shaped by a history of interactions stored in sensorimotor schemes from previous performances.

Perception based on coordinated motions radically changes the way the agent relates to the environment (Van Dijk and Withagen, 2016, p. 11). As Gibson also noticed, the perception of affordances seemed to have a distinctive temporal quality as well: “The feeling of past, present and future are merged or, more exactly, the activity of perception is acknowledged to be retrospective and prospective^2^” (Gibson, 1975, p. 300). Gibson suggested that the perception of affordances in the environment includes a non-successive experience of time; being of the present, but also being understood only in terms of the agent’s perceptual experiences (umwelt) and the action possibilities still open to it in the habitat. In other words, the concept of affordances makes reference to both the agent’s “past” abilities as well as to its “future” possibilities. Accordingly, what is inescapable, for human experience and action is not just the anticipatory aspect, but the full intrinsic temporality of the processes involved. A good model for this experiential part of affordances is the Husserlian analysis of the RIP structure of experience as Berthoz (2000) suggests, and as we saw in the last section, but we need a specific temporal scales proposal to analyze them from different angle, which will come in the next section.

Now, as we saw in section two, affordances can be seen as objective in the habitat or subjective as the umwelt for an agent, depending on the level of description. The umwelt is not stable either; it changes by the history of structural coupling the individual goes through in her ontogeny, as she goes actualizing affordances. In what follows, we will characterize the temporal scales that the umwelt (experiential affordances for a particular agent) has, claiming that the temporal integration of what constitutes the perception of affordances, offers a bridge to reconcile the “enactive” and the “ecological” descriptions of the agent-environment system in action.

### Scales for the Perception and Learning of Affordances

A number of theorists have proposed to capture the subpersonal processes that would instantiate this Husserlian structure shown in Figure 1 by using a dynamical systems approach (Van Gelder and Port, 1995; Varela, 1999; Thompson, 2007). On this view, action and our consciousness/experience of action arise through the concurrent participation of distributed regions of the brain and their sensorimotor embodiment which are established in three scales (Varela, 1999), the first two which are said to be directly relevant to protentional-retentional processes (Gallagher, 2013, p. 125; Gallagher, 2017a, p. 8):

(1) the *elementary* scale (the 1/10 scale varying between 10 and 100 ms): intrinsic cellular rhythms,
(2) the *integration* scale (the 1 scale, varying from 0.5 to 3 s): neuronal processes,
(3) the *narrative* scale involving memory (the 10 scale, more than 3 s).

To account for processes, enactivists appeal to the ideas of a dynamical system and diachronic constitution (Kirchhoff, 2015). Brain, body, and environment are said to be dynamically coupled in a way that forms a system, and the coupling is not equivalent to identity of material parts; rather it involves physical relational processes. Significant changes in one part of the system will cause changes or adjustments in the other parts. For the enactivists just these dynamical causal relations constitute the system. Because these processes occur on several timescales, it is helpful to introduce this three-fold distinction in temporal and dynamical registers.

It is important to note that as Beaton (2013, p. 300), when Varela says neural mechanisms, he means it: he supposes that it is the goings on in the brain that will directly correspond to the details of attention disclosed via phenomenology. And here we want to claim that these three temporal scales are all intertwined within the environmental constitutional situation that is involved in the particular embodied perception/experience of affordances. In other words, in the integration and in the narrative scale, the neuronal/sensorimotor processes are diachronically constituted by the dynamically coupled brain, body, and environment system; and this happens via the perception of affordances.

Through the integration and synchronization of the scales is that the perception of affordances are processed almost momentarily: there are aggregates formed that are impossible to comprehend but become complete sensorimotor schemes in the 1 scale: neuronal processes are integrated, which, at the neurophysiological level, involves the integration of cell assemblies all through the body. Phenomenologically, the integrative scale corresponds to the experienced living present (the umwelt), the level of a fully constituted cognitive operation; motorically, it corresponds to a basic action, for example, reaching, grasping, and as we saw in the previous section, it can also be characterized by the RIP structure.

Furthermore, from the enactive and the ecological perspective, perception of affordances is temporally extended, because differences in temporal scales are constitutive of sensorimotor enactive dynamics, and sensorimotor enactive dynamics are constitutive of the perception of affordances, hence perception of affordances is intrinsically temporal. And the three-fold distinction made by Varela is helpful for thinking how the two approaches can fit together. Thus, perception of affordances is constituted by the integration of the 2 first scales, and the experience of it has a RIP structure.

The time to complete the perception of affordances is not dependent on a fixed integration period as measured by a clock, it is dependent dynamically on the number and arrangement of cells assemblies that are contributing at that time in relation with the affordances that the subject is interacting with in a given coordination with the environment. That is why the integration scale is flexible, depending on the number of elements in the context and also with the corporal state and age, previous experiences, among others. On an enactivist interpretation, the primal impression is not the origin and point of departure but the “boundary” between retention and protention or the result of a dynamical interplay between retention and protention. The umwelt then is not simply a passive reception of the present; it enacts the present, it constitutes its meaning in the shadow of what has just been experienced (retention), and in the light of what it anticipates (protention) for a particular agent (Gallagher et al., 2017, p. 89).

In the case of action, the intrinsic temporality of the perception of affordances should be considered as pragmatically directed toward the meaningful possibilities the agent sees in the world. This lines up well with Husserl’s conception of embodied experience as an anticipatory “I can” which draws on my prior experience and my current state. As Husserl puts it, “every living is living toward.” This anticipatory intentionality is an apprehension of the possibilities or the affordances in the present. (Gallagher et al., 2017, p. 89)

The RIP structure is an account of how experience is constituted at the integrative scale from the perception of affordances: at the integrative scale, affordances are experienced as solicitations.

Summarizing until now, we can say that perception of affordances is constituted by the abilities and characteristics of the environment as relations that occur with an intrinsic temporality that is related to different temporal scales interconnected between them. This constitution is important because perception of affordances is then temporally extended and therefore can be characterized by the three-fold scale structure proposed by Varela. The two first scales are constitutive of the perception affordances whenever they are actualized and therefore whenever they bear an umwelt for the agent. But what about the third scale? We will now go into it.

The conception of affordances 2.0 is actually intended to provide a way of talking about affordances while acknowledging the fact that individual organisms learn:

The variety of niche construction sketched in Affordances 2.0 is an equally tightly coupled animal–environment system. It differs from the much-discussed biological case in two ways. First, the constructed niche is for an individual organism, not for a population. Second, it occurs over shorter time scales—an animal’s activities alter the world as the animal experiences it, and these alterations to the phenomenological-cognitive-behavioral niche, in turn, affect the animal’s behavior and the development of its abilities to perceive and act, which further alter the phenomenological-cognitive-behavioral niche, and on and on. Affordances 2.0, therefore, emphasizes the connections between radical embodied cognitive science and its natural allies in biology, that is, developmental systems and niche construction. (Chemero, 2009, p. 152)

Here, we are introducing *learning of affordances*, which go in the third temporal scale, the narrative one, in the explanation we are proposing. I want to maintain that perception (or perceptual experience) occurs at the integrative scale, and that what occurs at the narrative scale is different from perception (it is learning). This goes in line with Jacobs and Michaels (2007, p. 246) proposal that there is a short time scale of perceiving and acting and a longer time scale of learning, and here we propose that this timescale is the narrative scale in the three-fold distinction proposed by Varela. At this narrative scale, the affordances are seen as potentialities in the habitat (dispositions) in particular niches and become umwelts when they are actually perceived. There is a lot to say about learning, of course, but that is material for another paper. For the present purposes, perceptual learning can be defined as involving the increased ability to detect relevant affordances as the result of novel experience, as action capabilities change, and thereby affordances themselves change (Szokolszky et al., 2019, p. 8).

As I can see, these temporal scales can be a useful toolkit for explaining the perception and learning of affordances and at the same time unifying enactivism and ecological psychology claiming that affordances serve a different explanatory role depending on which time scale you consider them at. If you are interested in explaining the embodied assemblies that form the always changing sensorimotor contingencies, then you see the elementary scale. If you’re interested in explaining perception at the integrative scale, then affordances are solicitations. If you are interested in explaining change in the animal-environment system over developmental time, that is, learning time, then affordances are around what Chemero said they were when he proposed Affordances 2.0 and one should see at the narrative scale. But it is important to say that the three scales are always intertwined because learning and perception are ongoing processes that in many senses are impossible to separate.

Concluding, ecological psychologists can explain how the detection of perceptual information leads to the perception of particular affordances at the integrative scale, while the enactivist theory of sensorimotor agency can explain how an individual “selects” among perceived affordances in an embodied and situated way, according to her past experiences and learnings as a body-environment system (combining the elementary and narrative scales). As Baggs and Chemero say: “The ecological account of external structure is compatible with the enactivist account of the internal organization of the animal. Life happens in the dialectical confrontation of the two” (Baggs and Chemero, 2018, p. 14).

## Scale Matters

Living in an environment makes us adapt to the temporal structure of events (seen as the change in the layout of affordances, Chemero, 2000) or to the intrinsic timing of them that allow us (or not) to coordinate with certain affordances in a particular habitat. We could say from the last section that that both ecological psychologists and enactivists conceive of cognition as being inherently temporally extended and Varela’s three-fold distinction provides a useful toolkit for thinking about how the two approaches can fit together. Let’s expand on this.

In radical embodied science cognition needs to be understood in terms of the organism-environment system. On a dynamical systems interpretation, neuronal-level events on the elementary scale synchronize (by phase-locking) and form aggregates (the sensorimotor contingencies) that manifest themselves as incompressible but complete acts on the integrative scale (the solicitation of affordances). The narrative scale is meant to capture longer time periods that scale to complex actions and cognitive processes that may involve recollection, planning, intention formation, and so on, particularly, that scale could be understood in terms of learning. So, temporal scales matter because affordances serve a different explanatory role depending on which time scale you consider them at.

On the standard notion of synchronic constitution, subpersonal elementary scale neuronal processes constitute mental representational contentful processes that somehow scale up to experiences. All of the other factors (environmental, bodily, social, etc.) are causal but not constitutive. But in radical embodied cognitive science, mainly in enactivism and in ecological psychology, environment is constitutive because the constitution is diachronic, the temporal scales in which perception of affordances occur are relevant because that is what explains that cognition occurs as a system (agent-environment) and not only in the elementary scale, as cognitivism would claim. In other words, intentional action takes time, it begins and ends, and takes some duration in between. The temporal frame may vary (depending on the affordances performed) but they include all the environment-agent system and the three scales discussed above integrated and intertwined.

As Corris and Chemero say:

what exactly is the relationship between the fast and small events and the slow and large events? In both cases, the relationship must be such that events at the elementary scale are constitutive of events at integrative and narrative scales, but are also (in part at least) controlled by events at the integrative and narrative scales […] How are temporally extended events at the integrative and narrative scale able to influence the elementary-scale events that they are constituted by? How does recognizing an attractive lizard lead to the neural and muscular events involved in trying to catch it? (Corris and Chemero, 2019, p. 5)

Conceptualizing the structure of temporality of the perception of affordances serves to understand how this whole temporal window (the integration of the three temporal scales), which corresponds to the actual perception and learning of affordance(s) can be seen as a whole, and for epistemological reasons divided in temporal scales. Consequently, it might make sense to say that the entire organism-environment system retains changes in its structure as a result of the organism’s learning of sensorimotor contingencies that in turn give rise to (i) an explicit temporal perceptual experience given in terms of the perception of events, which are changes in the disposition of the potentialities of the world for different interactions with the agent (what Gibson, 1979, p. 12 called the ecological scale) and (ii) an implicit temporal perceptual experience in the sense of a coordinated activity of a specific type with a characteristic timescale (constitutive of sensorimotor schemes dynamics, the integrative scale). In other words, sensorimotor enactivism and ecological psychology can be understood complementarily in different temporal scales of the perception of affordances: the scale of the sensorimotor schemes dynamics and the scales of the solicitations of affordances.

In the terms of the proposal of Heras-Escribano (2019), this amounts to two levels of description of the interaction between a cognitive agent and her environment. These levels are also conflated to establish a bridge between enactivism and ecological psychology. For Heras Escribano, level 1 is the subpersonal level of networked systems that give rise to agency and enable behavior which comprises the relevant changes in the neurological, physiological, and chemical networks of the agent’s body. Level two, the personal level of analysis, refers to the organism-environment system that establishes a dynamical coupling thanks to ecological information, that is, through the available affordances. Level two would then be at the ecological scale, the scale about behavior: it explains how agents meaningfully interact with their environments (Heras-Escribano, 2016, p. 311). These two levels are not temporally separated, but mutually and simultaneously influence each other: “the perception of affordances may influence the pattering of habits due to [mastering] changes in sensorimotor contingencies and, at the same time, different sensorimotor contingencies may alter the movements that result from perceiving affordances” (Heras-Escribano, 2019, p. 26).

But here I would disagree in the conception that affordances are seen at the ecological scale and “influence the changes in sensorimotor contingencies,” because as I see it affordances are constituted by abilities (which are sensorimotor contingencies that get mastered and can change over time) and certain features of the environment. That is, they are relational and diachronically constituted by the scales we discussed before. Affordances as dispositions, which is implied in Heras Escribano’s levels, would only make sense if talking about the habitat for a species, but not for the actualization of perceived affordances for an individual, which would, as we have seen, involve an experience, the umwelt, and that allows to explain why some affordances solicit to some agents and not to others. So, the disagreement would be to understand the levels as separated, because as we have explained, differences in temporal scales are constitutive (not causal) of sensorimotor dynamics, and sensorimotor dynamics are constitutive of the perception of affordances.

Furthermore, as far as we can tell all enactivists are committed to granting a special status to the given umwelt and oppose the idea of perception as recovering a “pre-given” world (see, e.g., Varela et al., 1991; Thompson, 2007; Gallagher and Zahavi, 2012; Di Paolo et al., 2017; Gallagher, 2017a). So that the umwelt needs to be incorporated in the perception of affordances, we say it does through the temporal scales: the parameters of the sensorimotor system and the environment constrain each other in a dynamic, closed, and self-organized way, that is, they form autonomous and dynamic sensorimotor loops. These loops are called sensorimotor schemes and include all body and environmental structures that allow us to perceive affordances and execute a specific action.

Now, going back to Corris and Chemero’s question cited above, I would agree with them that synergies are a good and audacious answer to understand the relationship between the narrative scale and the elementary and the integrative. The basic idea is that the actualization of the affordance is what guides the processes in the first two scales, meaning that the causation would be macro-to-micro, and not otherwise: “if a behaving human is a synergy at the narrative scale, then the neural processes that partly compose that synergy are constrained by the behavior” (Corris and Chemero, 2019, p. 7). And this behavior is given through affordances. Van Orden et al. (2012) call it the “blue collar brain,” which claims that rather than being the executive in charge of the body, the brain does what is required by the activities of the embodied person. Synergies understood as “a functional grouping of structural elements (molecules, genes, neurons, muscles, etc.) which, together with their supporting metabolic networks, are temporarily constrained to act as a single coherent unit” (Kelso, 2009, p. 83), and there is empirical evidence that there are also human-tool synergies and interpersonal synergies (Dotov et al., 2010, 2017).

So, affordances would be synergies that are temporarily constituted as a whole in the different temporal scales we have developed here, with a recurrent RIP structure in the experience scales (the integrative). “Synergies self-organize apace with the flow of context and behavior. This is sufficient to update on-going constraints that anticipate the requirements for oncoming behavior. Invariant or smoothly changing aspects of the world yield invariant or smoothly changing constraints at a pace that is slower than brain dynamics. These constraints inform behavior by limiting the degrees of freedom about what can happen next, leaving open the possible kinematic changes that the body may enact in behavior” (Van Orden et al., 2012, p. 9). Finally, affordances should be characterized in terms of dynamical agent-environment systems, with abilities and aspects of the environment understood as constraints on the potential trajectories of such systems. Affordances, then, can be conceived as possible states of agent-environment systems, or as particular locations within the overall state space of these systems.

## Conclusion

Radical embodied cognitive science, mainly sensorimotor enactivism and ecological psychology, share many stands. But they also have many tensions. There are many resolutions of these issues, one of them, I suggest, involves taking the temporal dimension of affordances seriously, particularly in terms of interaction across multiple temporal scales. We characterized its intrinsic temporal structure borrowing the husserlian RIP *präsenzzeit* for the experience of the perception of affordances. Also, taking affordances as relations between abilities and features of the environment (affordances 2.0) we developed the temporality of the perception of them using Varelas’ three-fold model and Gallagher’s later use of it, but focusing on the dynamics of the agents’ embodied action, meaning to include the environment as a constitutive part of cognition genuinely. Behavior through affordances bears an experience with an intrinsic temporality that has a RIP structure.

I appealed to unify enactive and ecological approaches to cognitive science by emphasizing the fact that both approaches view cognitive processes as being inherently temporally extended, and my hypothesis is that characterizing the temporal scales in which perception of affordances occur, they can serve different purposes of explanation within the theories. The central claim of the present paper is that affordances should be understood in terms of these three different timescales. I showed that these temporal scales can be a useful toolkit for explaining the perception and learning of affordances and at the same time unifying enactivism and ecological psychology claiming that affordances serve a different explanatory role depending on which time scale you consider them at. For this, I think that perception of affordances should be characterized in terms of dynamical agent-environment systems, with abilities and aspects of the environment (affordances 2.0) understood as constraints on the potential trajectories of such systems. These constraints or parameters are themselves modifiable over time, in terms of both the development of abilities and the shaping and informing of the material world. Abilities and situations in the environment (affordances 2.0) are not the same the first time one engages with it than the second, third time, etc. This has as a consequence that the repetition of the perception of affordances has an impact on the other temporal scales, mostly because of neuronal facilitation and plasticity in the sensorimotor schemes: when one masters an ability, the sensorimotor scheme occurs faster (in the elementary and integrative scales) than the first times one performed it. Likewise, in the narrative scale the tempos move, because expectancy and anticipation are transformed (one already expects what will happen and knows the umwelt about it).

Finally, sensorimotor enactivism and ecological psychology can be used as complementary explanations through the scales of the perception of affordances: while ecological psychology focuses on explaining the dynamics at the level of the organism-environment system (the ecological gibsonian scale, the narrative and integration temporal scales), sensorimotor enactivism focuses on explaining the dynamics at the intra-organismic level (the elementary and integrative scales). But, remarking it again, the three temporal scales constitute the perception of affordance(s), where the scales are always integrated and intertwined. Likewise, these temporal scales can be a useful toolkit for explaining the perception and learning of affordances and at the same time unifying enactivism and ecological psychology claiming that affordances serve a different explanatory role depending on which time scale you consider them at. If you are interested in explaining the embodied assemblies that form the always changing sensorimotor contingencies, then you see the elementary scale. If you’re interested in explaining perception at the integrative scale, then affordances are solicitations. If you are interested in explaining change in the animal-environment system over developmental time, that is, learning time, then affordances are around what Chemero said they were when he proposed Affordances 2.0 and one should see at the narrative scale. But it is always important to say that the three scales are always intertwined because learning and perception are ongoing processes that in many senses are impossible to separate.

This also has a consequence that ecological psychology can explain how the detection of information in a habitat leads to the perception of particular affordances for a particular species, meanwhile the enactivist theory of sensorimotor agency can account for variations in perception-action of affordances at the level of the individual organism, explaining how certain affordances get a soliciting character because of previous experiences and through the particularities of the sensorimotor schemes dynamics which permit them to actualize. This is because perception brings forth an experience, so it might make sense to say that the entire organism-environment system retains changes in its structure as a result of the organism’s learning of sensorimotor contingencies that in turn give rise to (i) an explicit perceptual experience given in terms of the perception of events, which are changes in the disposition of the potentialities of the world for different interactions with the agent (the ecological scale) and (ii) an implicit perceptual experience in the sense of a coordinated activity of a specific type with a characteristic timescale (constitutive of sensorimotor schemes dynamics). In other words, sensorimotor enactivism and ecological psychology can be used as complementary explanations for different temporal scales in the perception of affordances.

Describing cognitive processes as being constituted in this temporally integrated dynamic system could also be very helpful in explaining more precisely the relationship between cognition and action with the toolkit of the temporal scales. Practically, I think this way of thinking could promote research that systematically varies a hierarchy of time scaled contexts, both for ecological psychology and for sensorimotor enactivism.

## Author Contributions

MG wrote the initial draft of the manuscript; after discussion and recommendations from Sergio Martinez and colleagues at the Workshop Ecological Psychology and Enactivism MG wrote the final draft.

## Conflict of Interest

The authors declare that the research was conducted in the absence of any commercial or financial relationships that could be construed as a potential conflict of interest.

## Footnotes

1 According to Gallagher (2013, p. 138) the term simply denotes that “the experiencing (sensing) act of consciousness [is] temporally extended.”

2 It is important to notice here that the retrospective and prospective character of affordances noticed by Gibson can now be retentional and protentional, borrowing Husserl’s terminology. This can be done as I have showed that experience happens as perception does, and perception is of affordances. Experience does not belong to a metaphysical realm, but to perception itself.
